# Giant intrathoracic mycotic aneurysm of left subclavian artery

**DOI:** 10.4103/0971-5851.73592

**Published:** 2010

**Authors:** R. P. Bansal, Prerna Gupta, Lalit M. Sharma

**Affiliations:** *Department of Radiodiagnosis, Bhagwan Mahaveer Cancer Hospital and Research Centre, JLN Marg, Jaipur, India*; 1 *Department of Medical Oncology, Bhagwan Mahaveer Cancer Hospital and Research Centre, JLN Marg, Jaipur, India*

**Keywords:** *Aspergillosis*, *computed tomographic angiography*, *giant mycotic aneurysm*, *subclavian artery*

## Abstract

Fungal infections are serious and important cause of morbidity and mortality in immunocompromised patients. Angioinvasive aspergillosis causing mycotic aneurysm of major blood vessels is very rare and only a few cases are reported in literature. We hereby report one case of acute lymphoblastic leukaemia presenting with this fatal complication.

## INTRODUCTION

Mycotic aneurysms of subclavian artery, caused by angioinvasive aspergillosis, in immunocompromised children are very rare. Only one case has been reported previously to the best of our knowledge.[1]

We present a case of 6-year-old girl undergoing chemotherapy for acute lymphoblastic leukemia, who presented to us initially with pneumothorax and then rapidly developed a fatal giant intrathoracic pseudoaneurysm of left subclavian artery over a period of 1 month, due to infection with angioinvasive aspergillosis. Serial chest radiographs, computed tomography (CT) and computed tomography angiography were performed to establish the diagnosis and follow the course of the disease which eventually had a fatal outcome.

## CASE REPORT

A 6-year-old girl, a diagnosed case of acute lymphoblastic leukemia on chemotherapy in another hospital, was admitted to our hospital on 11 August 2008, with complaints of fever and cough for the last 15 days, not responding to antibiotics.

Her blood examination on the day of admission showed total leukocyte count of 4100/mm^3^, platelet count of 41,500/mm^3^ and Hb of 6.3 g/dl.

**Figure 1:**
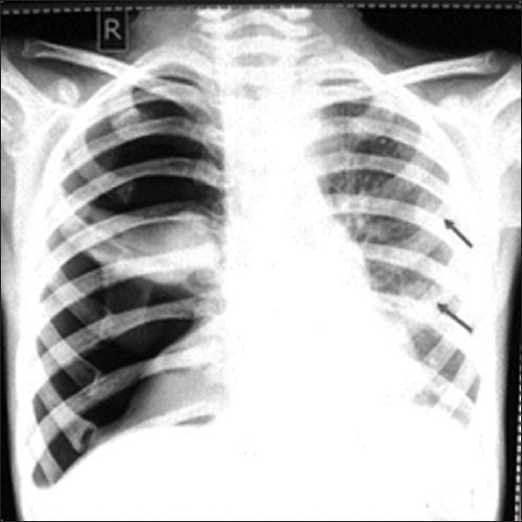
Chest radiograph showing right-sided tension pneumothorax and multiple cavitating nodules in left lung (arrows)

Chest radiograph [Figure 1] done on the day of admission revealed right-sided tension pneumothorax with collapse of underlying lung. Left lung showed multiple small cavitating nodules. Intercostal drainage tube was inserted and her pneumothorax completely resolved and the underlying lung completely expanded.

**Figure 2:**
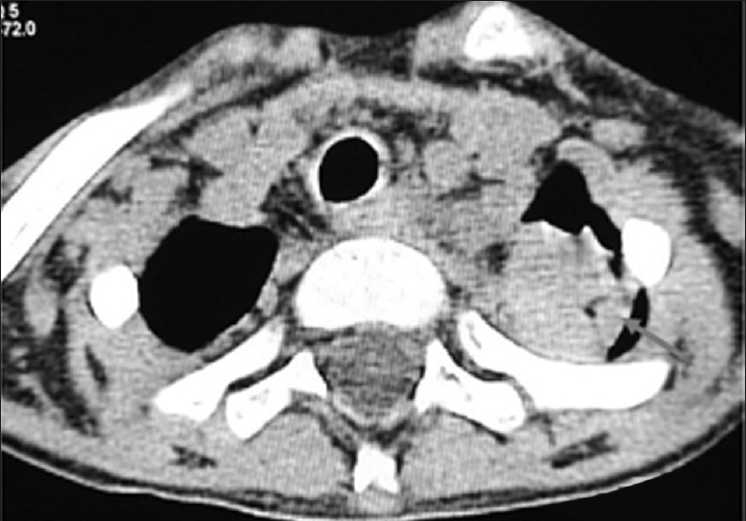
HRCT chest (mediastinal window) showing left apical mass with air crescent sign (arrow) and mediastinal invasion

**Figure 3:**
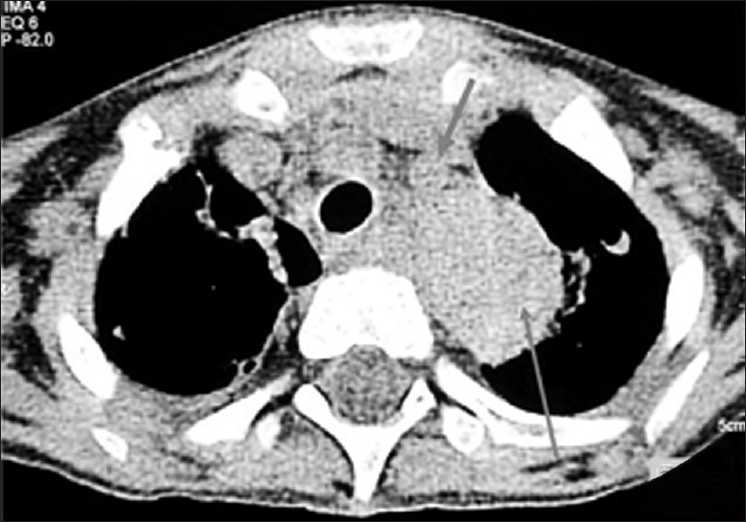
HRCT chest (mediastinal window) showing left apical mass (long arrow) at a lower level with engulfment of left subclavian artery (thick, short arrow)

**Figure 4:**
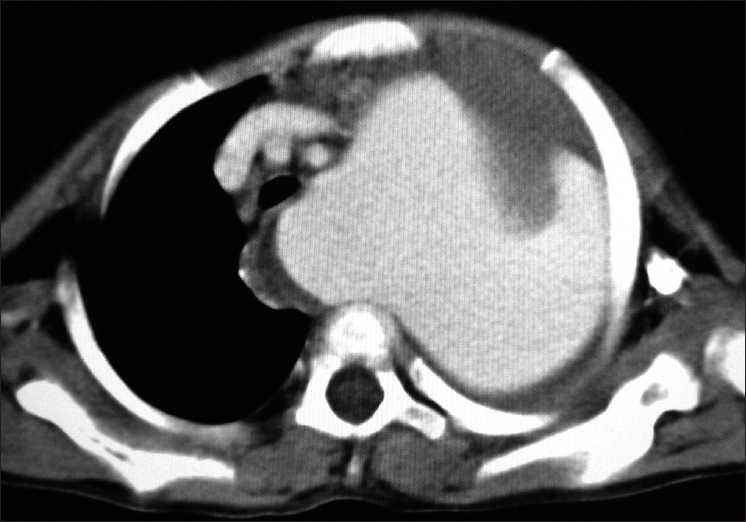
HRCT chest (lung window) showing multiple nodules, halo sign (thick, short arrow) and pneumatoceles (thin, long arrow)

High-resolution computed tomography (HRCT) of chest, done 3 days later on 14 August 2008, showed left apical small soft tissue density mass with air crescent inside [Figure 2], invading into mediastinum. Left subclavian artery was not visualized, suggestive of engulfment by the mass [Figure 3]. In addition, multiple nodules, some of them showing cavitations and peripheral halo, small peripheral consolidations and thin-walled pneumatoceles, were also seen in both lungs [Figure 4]. Considering these CT features, a presumptive diagnosis of angioinvasive aspergillosis was made. Presence of multiple thin-walled pneumatoceles was considered to be due to concurrent staphylococcal infection with rupture of one of the pneumatoceles leading to initial right-sided pneumothorax. Patient was treated with Amphotericin B and Vancomycin for a week, to cover aspergillosis as well as staphylococcal infection.

**Figure 5:**
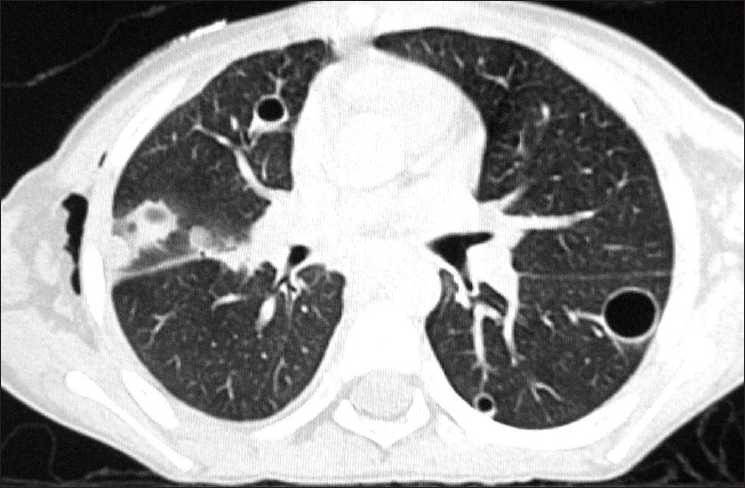
CECT chest (mediastinal window) showing giant pseudoaneurysm (thin, long arrow) arising from outer wall of left subclavian artery (thick, long arrow). Left, Common Carotid Artery (CCA) and right innominate artery are marked by short arrows

Her fever subsided; however, cough and respiratory distress persisted. Serial chest radiographs showed a homogenous left upper zone mass with well-defined lower margin which increased in size to almost completely occupy the left hemithorax. A contrast-enhanced CT (CECT) [Figure 5] done on 12 September 2008, about a month later, showed giant false aneurysm arising from proximal part of left subclavian artery, almost completely occupying left hemithorax with collapse of underlying lung and contralateral mediastinal shift.

**Figure 6:**
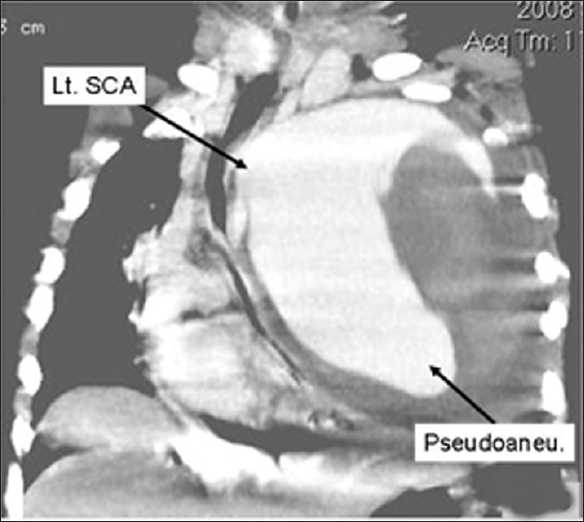
CT angiography (coronal reconstruction) showing large pseudoaneurysm arising from outer wall of left subclavian artery

CT angiography [Figure 6] confirmed the diagnosis of left subclavian artery giant pseudoaneurysm with large thrombus inside it.

She succumbed to her illness and died while on her way to higher center for endovascular management of aneurysm.

## DISCUSSION

Mycotic aneurysms of subclavian artery caused by angioinvasive aspergillosis are very rare and only a few cases are reported in literature.[1]

Angioinvasive aspergillosis is a serious infection frequently seen in immunocompromised patients. It is characterized on CT by nodules surrounded by ground glass halo (halo sign) or pleural based wedge-shaped areas of consolidation. Separation of necrotic fragment from adjacent lung parenchyma results in formation of air crescent sign.[2]

The halo sign represents hemorrhage around the central infarctions caused by angioinvasion.[3] Cavitation of a nodule usually coincides with recovery of neutrophil count.[4]

Visrutaratna *et al*.[1] reported a similar case of left subclavian artery aneurysm in a 9-year-old girl with acute lymphoblastic leukemia, having completed her chemotherapy and subsequently presenting with febrile neutropenia and mild hemoptysis due to angioinvasive aspergillosis.

Saliou *et al*.[5] reported a case of mycotic aneurysm of left subclavian artery in a 32-year-old man having leukemia, who presented with recurrent hemoptysis which was caused by fistulization of lung parenchyma.

The various mechanisms postulated in the development of mycotic aneurysms include: embolism of vasa-vasorum, direct wall invasion and erosion of the vessel from an adjacent lesion of the lung or via lymphatic system.[5]

Very few cases of aspergillosis induced mycotic aneurysms of sites other than subclavian artery[1,5] in children are reported. They include two cases involving descending aorta,[6] one case of common carotid artery[7] and another case of aortic arch.[8]

In our case also, the patient was immunocompromised and had typical CT features of angioinvasive aspergillosis. She presented with fever and pneumothorax (presumably due to rupture of pneumatocele caused by concurrent staphylococcal infection). Her aneurysm progressed rapidly over a course of 1 month to completely occupy left hemithorax and ultimately proved to be fatal.

We report this case for its rarity and to alert the readers that in appropriate clinical setting of immunocompromised state with CT features of aspergillosis, a strict vigil should be kept over the patient regarding development of mycotic aneurysm so that timely intervention can be done.
